# Enhancing Mid‐Upper Facial Contours: Hairline Transplant Solutions for East Asian Women With High and Wide Foreheads

**DOI:** 10.1111/jocd.70374

**Published:** 2025-08-04

**Authors:** Wei Wu, Chi Liu, Peiqi Zhang, Shunuo Zhang, Qian Liu, Meihua Di, Xiaohui Yang, Siyuan Zhu

**Affiliations:** ^1^ Department of Plastic and Reconstructive Surgery, Ninth People's Hospital Affiliated to Medical School Shanghai Jiaotong University Shanghai China; ^2^ Department of Plastic Surgery Second People's Hospital Shanghai China; ^3^ Department of Surgery Sixth Hospital Fuzhou China; ^4^ Department of Plastic Surgery Ruichang People's Hospital Ruichang China; ^5^ Department of Medical Aesthetics Dongtai City Hospital of Traditional Chinese Medicine Yancheng China

**Keywords:** hairline transplant, high and wide foreheads, mid‐upper facial contours

## Abstract

**Background:**

A high and wide forehead is a common congenital facial characteristic among East Asian women, often resulting in an imbalanced mid‐upper facial proportion, diminished three‐dimensional contour, and reduced femininity. Although this facial morphology is often congenital, such features frequently lead to appearance‐related anxiety, affecting patients' confidence and social interactions.

**Objective:**

This study aimed to investigate how a personalized hairline design strategy using follicular unit extraction (FUE), tailored to East Asian facial proportions, can enhance mid‐upper facial contours in women with high and wide foreheads.

**Methods:**

A total of 60 female patients with high and wide foreheads were enrolled, with a mean age of 33.3 ± 7.1 years. Preoperative measurements showed a mean mid‐frontal height of 7.48 ± 0.46 cm and a mean intertemporal width of 14.66 ± 0.65 cm. On average, 3243 ± 1038 follicular units were transplanted at a recipient density of 50–60 FUs/cm^2^. Postoperative assessments included improvements in forehead contour, naturalness of the hairline, and documentation of adverse events.

**Results:**

The average reduction in mid‐frontal height was 1.33 ± 1.10 cm, and the intertemporal width was reduced by 1.47 ± 0.57 cm. On a 5‐point Likert scale, the average satisfaction score for mid‐upper facial contour improvement was 4.70, while the score for the overall hairline appearance was 4.52. Only minor postoperative discomfort was reported, with no severe complications observed.

**Conclusion:**

Hair transplantation via FUE is an effective approach for improving mid‐upper facial proportions in East Asian women with high and wide foreheads, resulting in a softer and more three‐dimensional forehead contour. Surgical design based on East Asian facial and hair characteristics yields high patient satisfaction and favorable safety outcomes, supporting its broader clinical application.

## 1 Introduction

A high and wide forehead is a common concern in female facial aesthetics, often resulting in disproportionate facial features that reduce the overall three‐dimensionality and femininity of the face. Vertically, the face can be divided into upper, middle, and lower thirds, with a relatively smaller upper third being considered more aesthetically pleasing in women [1, 2]. When the length of the forehead exceeds that of the middle and lower thirds, it is typically classified as a “high forehead,” which disrupts facial harmony and can impart an older or even more masculine appearance [3, 4]. East Asian populations are generally characterized by brachycephalic facial structures, with prominent zygomatic bones, a low nasal bridge, and a broad midface [5]. Although women with high and wide foreheads typically do not experience significant hair loss, they often suffer from aesthetic concerns such as appearance‐related anxiety, challenges in achieving satisfactory makeup coverage, and heightened psychological burden due to congenital frontal structural disproportion, all of which can negatively impact social confidence and overall quality of life [3].

Therefore, optimizing the vertical proportions and lateral width of the mid‐upper face (mid‐upper face refers to the upper two‐thirds of the face, spanning from the frontal hairline to the level of subnasale) is essential for enhancing overall facial softness and three‐dimensionality. Forehead contouring procedures have emerged as a means for individuals seeking to refine facial curvature or address psychosocial distress associated with less traditionally feminine facial features [6]. In recent years, hairline‐lowering procedures have gained popularity among young East Asians, as a shorter forehead and lower hairline are perceived to be more aesthetically desirable [7]. Although forehead reduction surgery is increasingly recognized as a safe and effective means of improving frontal contour in women, it carries risks such as scalp numbness, postoperative telogen effluvium, and visible linear scarring [3, 8, 9, 10]. Compared to forehead reduction surgery, hair transplantation not only reduces forehead height but also narrows the forehead by adjusting the lateral hairline, without resulting in linear scarring. Moreover, patient satisfaction with hairline correction procedures has significantly improved [5, 11].

**FIGURE 1:**
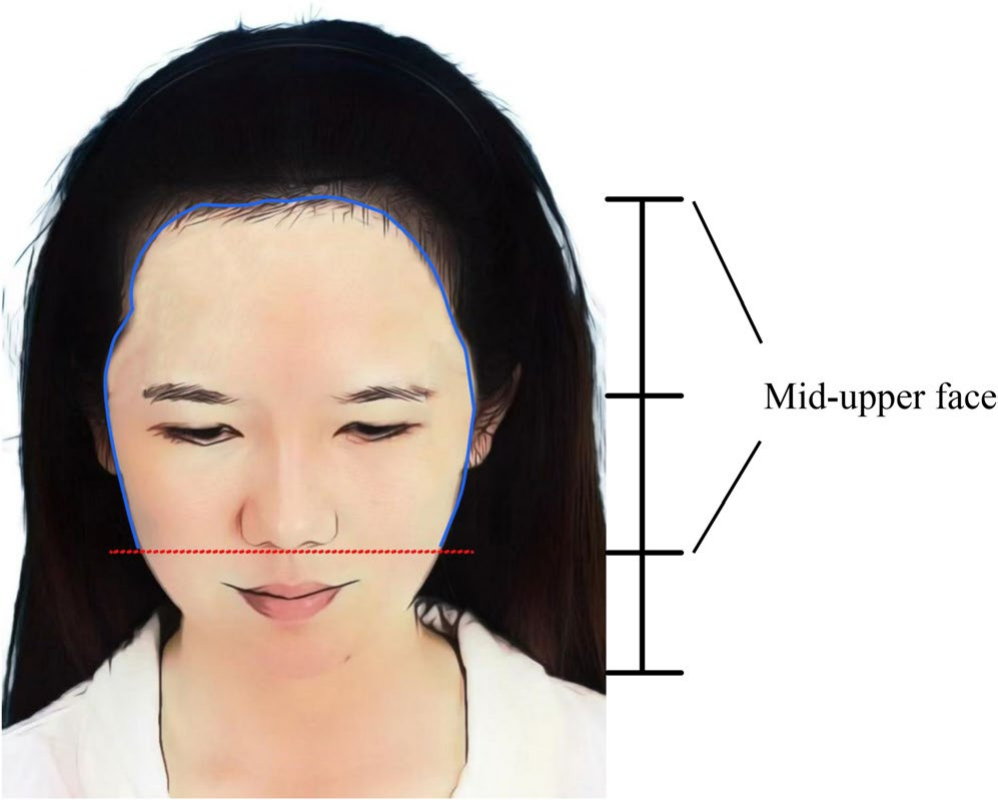
Illustration of the mid‐upper face and mid‐upper facial contour. The mid‐upper face refers to the upper two‐thirds of the face, spanning from the frontal hairline to the level of the subnasale. The red line represents the level of the subnasale, while the blue line outlines the mid‐upper facial contour, which is the target region for aesthetic optimization through hairline transplantation in East Asian women with high and wide foreheads.

This study systematically evaluates the aesthetic efficacy of hairline transplantation in enhancing the mid‐upper facial contour of Asian women with high and wide foreheads (Figure 1). By analyzing patient satisfaction and postoperative safety, we propose a personalized, zone‐based hairline design framework tailored to this population. We hypothesize that a customized hairline design—focusing on frontal height reduction, frontotemporal enhancement, and temporal peak repositioning—can effectively improve the aesthetic balance and perceived femininity of the mid‐upper face in East Asian women with high and wide foreheads.

## 2 Data and Methods

### 2.1 Participants

This study was a retrospective analysis that included a total of 60 female subjects, who underwent FUE hair transplantation between May 2018 and February 2024. The inclusion criteria were as follows: female patients with high and wide foreheads; patients aged from 18 to 60 years old without obvious hair loss manifestation for at least half a year; adequate density of hair in the donor area. The exclusion criteria were the presence of scalp lesions affecting the surgical operation, such as acute or chronic scalp infections, scarring alopecia, and so on; combined severe mental disorders or those who do not cooperate with the treatment; past history of serious systemic diseases, such as autoimmune diseases, endocrine disorders (such as uncontrolled thyroid dysfunction and polycystic ovary syndrome), coagulation disorders, or keloidal body; pregnant or lactating women. The study strictly followed the ethical guidelines related to the Declaration of Helsinki, and all patients signed an informed consent form before surgery.

### 2.2 Surgical Techniques

#### 2.2.1 Preoperative Hairline Design

**FIGURE 2:**
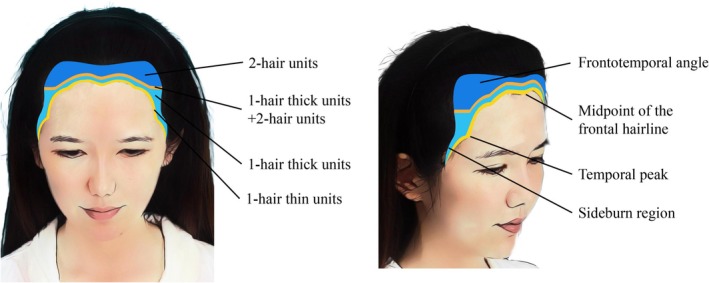
Hair design plan for female patients with high and wide foreheads. This design is for female patients with high and wide foreheads who do not exhibit significant hair loss. The natural transition of the hairline is the key to overall aesthetics, and the foremost edge is implanted with 1‐hair thin units to create a soft, natural wavy female hairline (Yellow area). The transition zone immediately behind the hairline, approximately 2 cm wide, is divided into two parts. 1‐hair thick units are implanted in the front half (Light blue area), and a mixture of 1‐hair thick units and 2‐hair units are implanted in the back half (Orange area) to achieve a natural transition effect. In order to enhance the visual thickness, 2‐hair units are implanted in the frontotemporal angle and the area where the hair meets normal hair to further enhance the overall fullness (Dark blue area). The key areas for female hairline transplantation include the frontotemporal angle, midpoint of the frontal hairline, temporal peak, and sideburn areas.

**FIGURE 3:**
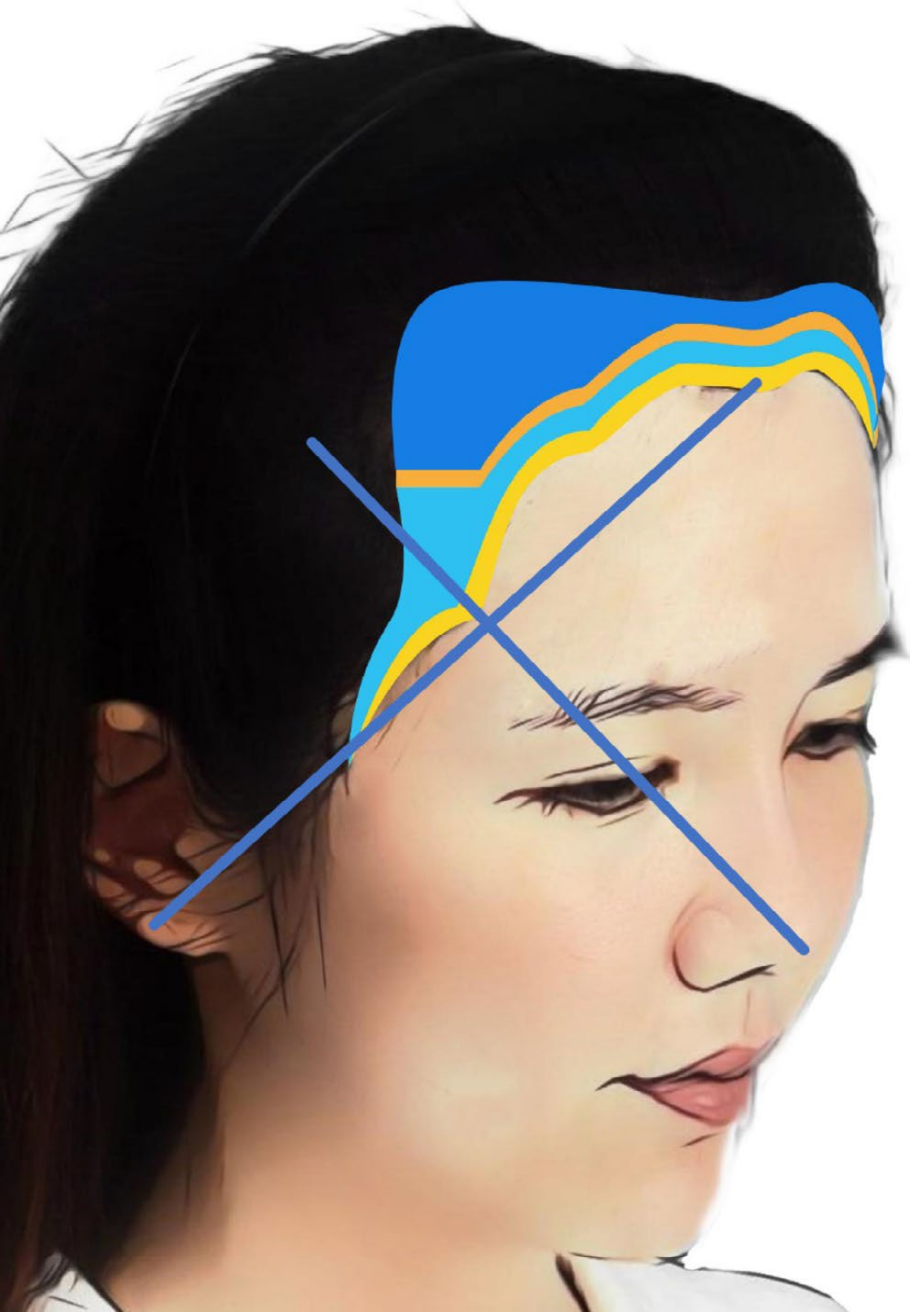
Anatomical reference lines for locating the temporal point. The temporal point was determined by the intersection of two anatomical reference lines. The first line was drawn from the nasal tip through the midpupil of the ipsilateral eye. The second line extended from the earlobe to the midpoint of the frontal hairline. The intersection of these two lines approximates the optimal position of the temporal point.

Before surgery, a personalized implantation plan is formulated based on a comprehensive assessment of the hairline position, forehead height, and width. The natural transition and gentle curve of the female hairline are crucial to the overall facial harmony. 1‐hair thin units are implanted at the most anterior edge of the hairline. The buffer zone behind the leading edge of the hairline, approximately 2 cm wide, is divided into two parts. 1‐hair thick units are planted in the front part, and a mixture of 1‐hair thick units and 2‐hair units are planted in the back part. To enhance the visual density, 2‐hair units are planted in the frontotemporal angle and the area that borders the native hair. Key areas for female hairline implants include the frontotemporal angle, the midpoint of the frontal hairline, the temporal peak area, and the sideburn area (Figure 2). To determine the position of the temporal peak point, two anatomical reference lines were used: one line drawn from the tip of the nose through the midpupil, and another from the earlobe to the midpoint of the frontal hairline. The intersection of these two lines defines the optimal location for the temporal peak (Figure 3). This approach helps restore lateral symmetry, narrows the perceived width of the midface, and contributes to a more feminine facial appearance.

#### 2.2.2 Harvesting and Preparation of Follicular Units

**FIGURE 4:**
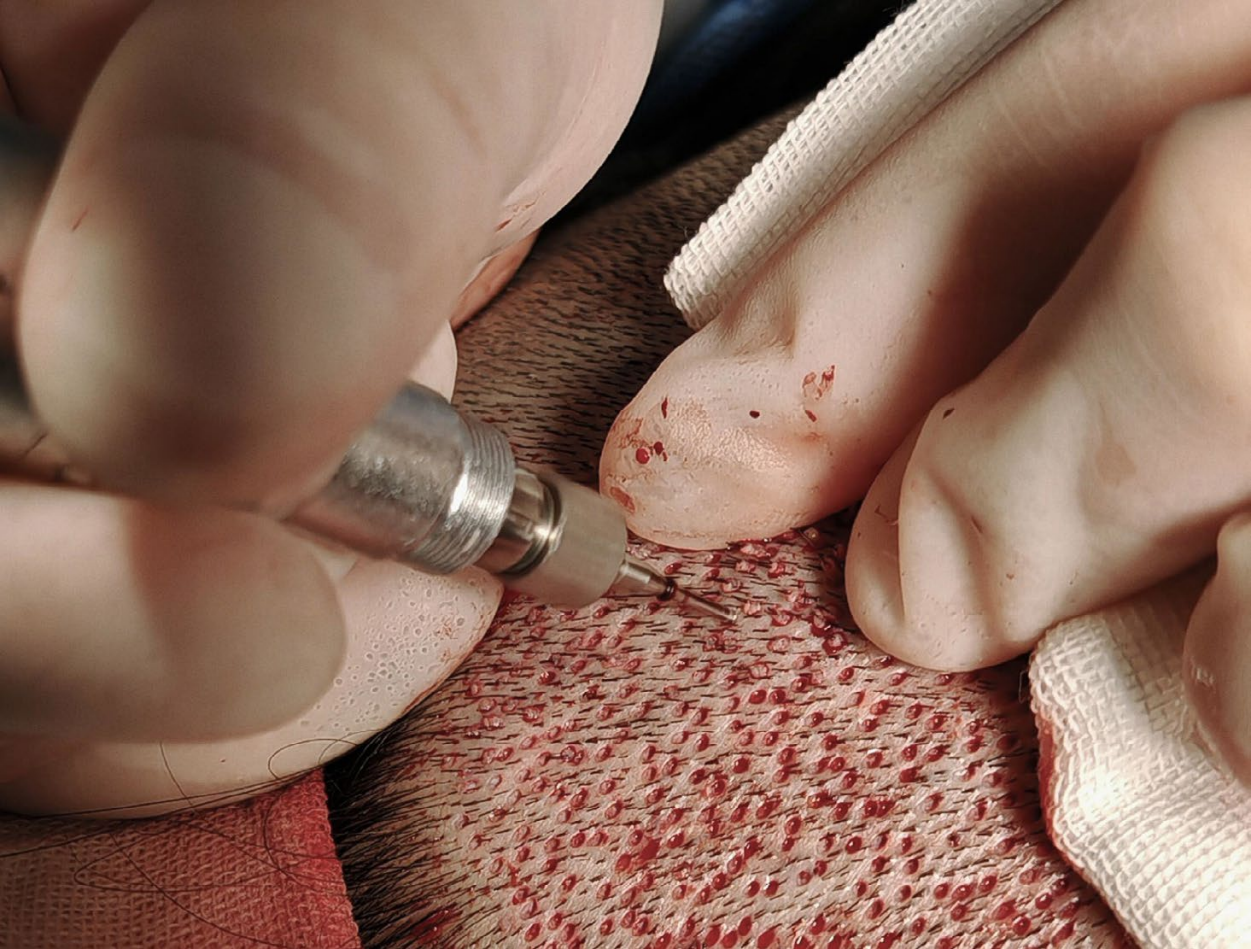
Extraction of follicular grafts. The scalp is retracted with an assisting hand during the extraction process, and the extracted grafts need to be kept intact.

In this study, all female patients had healthy hair in the posterior occipital region as the donor area. Hair in the donor area was trimmed to a length of approximately 1–2 mm before surgery, and the patients were placed in the prone position to ensure intraoperative comfort and stability. After routine disinfection and toweling, local infiltration anesthesia with 1% lidocaine (containing 1:200 000 epinephrine) was applied to the donor area. Follicular units were extracted using the FUE (follicular unit extraction) technique with a motorized, hybrid 0.7 mm punch (Figure 4). The punch is flared (trumpet‐shaped) with a sharp outer edge for scoring the epidermis and a dull inner edge for blunt dissection, reducing the risk of follicular transection [12]. 1‐hair, 2‐hair, and 3‐hair follicular units were harvested. Immediately after extraction, the follicular units were placed in a pre‐prepared Ringer's solution at 4°C–10°C in order to maintain their biological activity.

**FIGURE 5:**
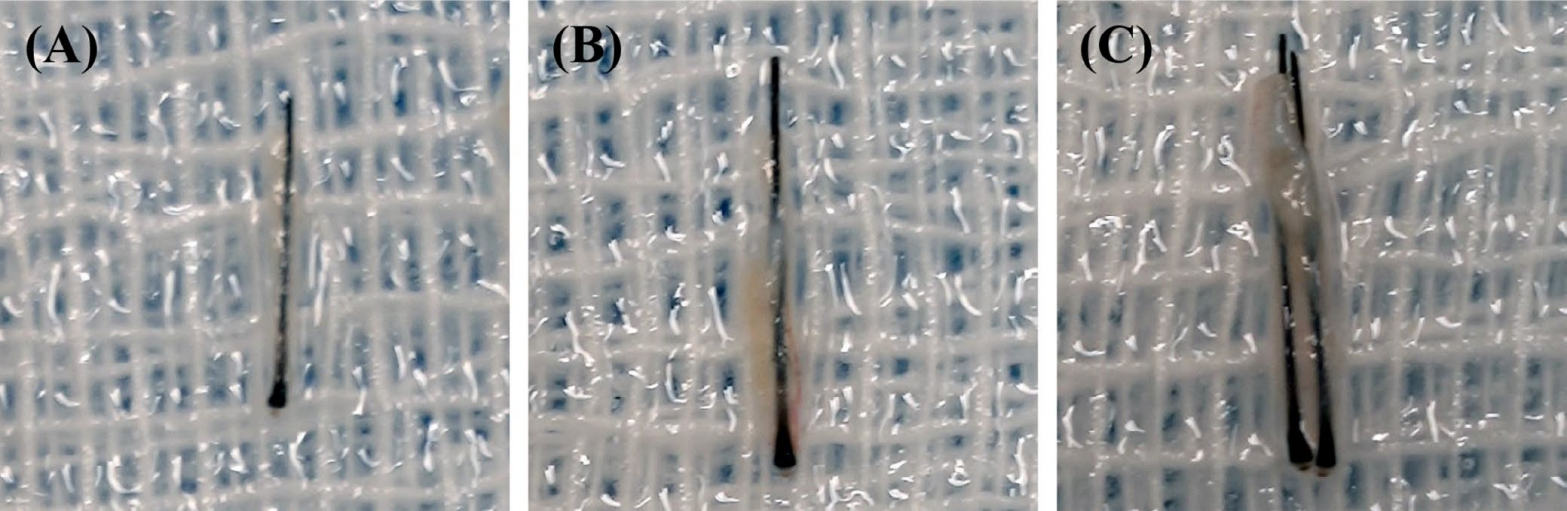
Different types of follicular units in the hair transplant surgery. (A) 1‐hair thin unit. (B) 1‐hair thick unit. (C) 2‐hair unit.

The harvested grafts were then trimmed under a 3× magnifying loupe. 3‐hair grafts were carefully trimmed and split under magnification into 1‐hair and 2‐hair units as needed. The operator classified the follicular units into 1‐hair thin unit, 1‐hair thick unit, and 2‐hair unit according to the number of hairs and thickness characteristics (Figure 5). All sorted hair follicle units were placed separately in graft storage plates, clearly marked with numbers, and continued to be immersed in 4°C–10°C Ringer's solution for backup.

#### 2.2.3 Implantation of Follicular Units

**FIGURE 6:**
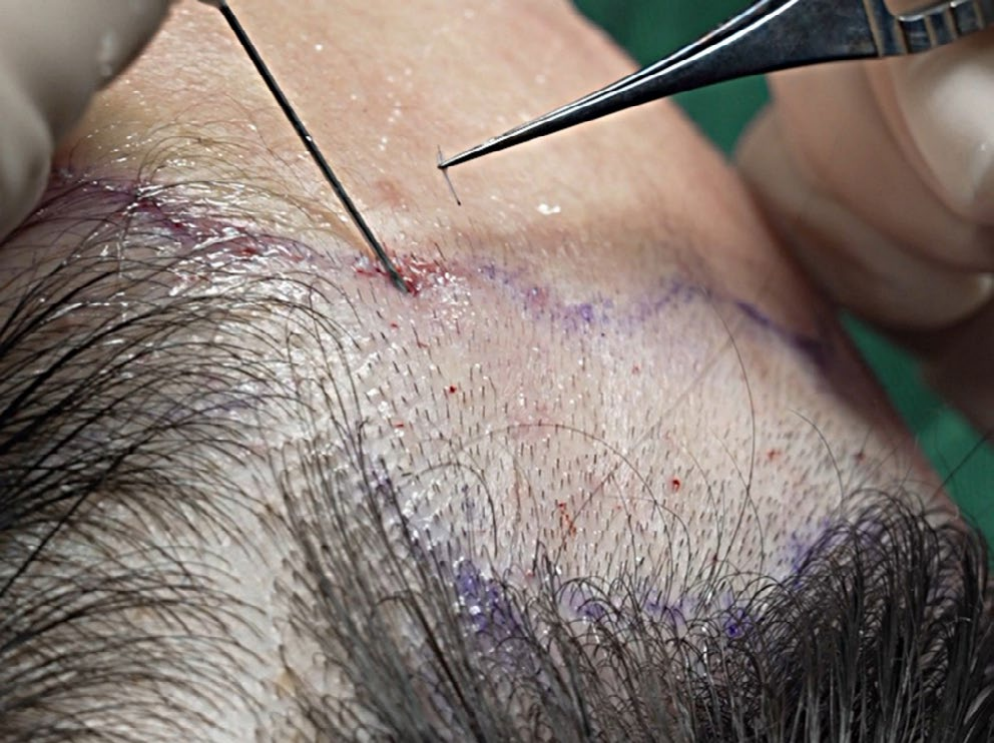
The implantation procedure of the follicular units. The grafts were implanted with the stick‐and‐place technique, in which each follicular unit was inserted immediately after the recipient site was created.

The recipient area was trimmed before implantation to improve visibility and accuracy during the placement of follicular units. In the process of follicular unit implantation, the operator adopted the stick‐and‐place technique, in which hair follicles were implanted immediately after the creation of the recipient sites (Figure 6). Syringe needles of 20G to 23G were used for the creation of the recipient sites, and the needle type was selected according to the type of hair follicle: 20G to 21G for 2‐hair units, 22G for 1‐hair thick units, and 23G for 1‐hair thin units. The implantation density of follicular units was approximately 50–60 units/cm^2^, depending on the area of the affected area and aesthetic goals. The direction of implanted hair follicles should be consistent with the natural growth direction of the original hair in the recipient area or designed with reference to the direction of normal scalp hairs. In order to achieve a natural effect, the implantation angle of the lateral hairline is generally at an angle of 10°–15° from the scalp, while the implantation angle of the frontal hairline area is mostly 15°–25°. The specific implant angle should be personalized according to different areas and individual patient differences in order to obtain the best natural post‐operative appearance.

### 2.3 General Characteristics

**FIGURE 7:**
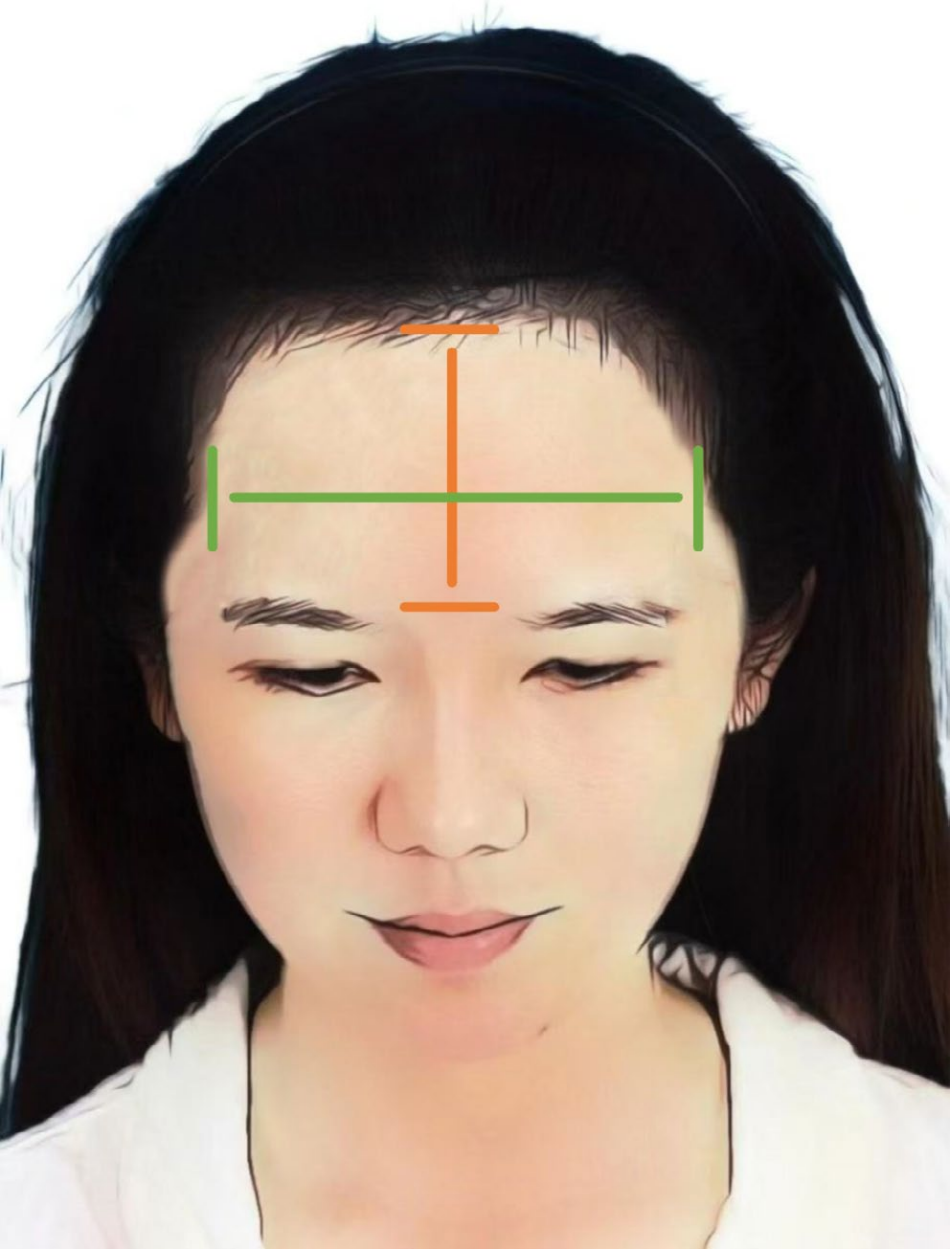
Measurement of the mid‐frontal line and intertemporal line for evaluating mid‐upper facial proportions. The mid‐frontal line was defined as the vertical distance from the glabella to the midpoint of the frontal hairline, representing the vertical height of the upper forehead. The intertemporal line was measured as the horizontal distance between the bilateral temporal points, reflecting the transverse width of the mid‐upper face.

Baseline characteristics were recorded following patient enrollment, including the number of participants, age, the length of the mid‐frontal line(the central vertical line on the forehead extending upward from the glabella to midpoint of the natural hairline) and intertemporal line(the horizontal distance between the left and right temporal points) [13] (Figure 7). These two anthropometric indicators were used pre‐ and post‐operatively to assess how hairline design and transplantation improved facial proportions in Asian women with high and wide foreheads.

### 2.4 Basic Information of Hair Transplant Surgery

The basic parameters related to hair transplantation were recorded in detail during the surgery. Specific data included the average number of transplanted follicular units (FUs), implantation density in the recipient area, average duration of surgery, reduction in the mid‐frontal hairline, and narrowing of the intertemporal distance. Each of these variables was documented separately for subsequent analysis.

### 2.5 Postoperative Satisfaction Evaluation

To assess long‐term postoperative satisfaction, a structured questionnaire was administered to the patients at least 6 months after surgery. Satisfaction was rated on a 5‐point Likert scale ranging from 1 to 5 (1 = very dissatisfied, 2 = dissatisfied, 3 = neutral, 4 = satisfied, and 5 = very satisfied.).

Participants were asked to respond to the following questions:

1. How satisfied are you with the improvement in your mid‐upper facial contours?
2. How satisfied are you with the overall appearance of your hairline after the transplant?

### 2.6 Safety Assessment

Postoperative adverse events in the donor and recipient areas were systematically assessed. Adverse reactions such as low graft survival rate, postoperative infection, itching of the scalp, localized swelling, noticeable scarring, bleeding at the surgical site, pain or discomfort, folliculitis, and improper hair growth direction were continuously monitored and recorded during the study period.

### 2.7 Statistical Analysis

Statistical analysis of all data in this study was performed with the help of SPSS software version 25.0. Quantitative data were expressed as mean ± standard deviation (SD).

## 3 Results

### 3.1 General Characteristics

A total of 60 female participants with high and wide foreheads were enrolled in this study. The mean age was 33.3 ± 7.1 years. The average distance of the mid‐frontal line was measured at 7.48 ± 0.46 cm, and the mean intertemporal distance was 14.66 ± 0.65 cm (Table 1).

**TABLE 1:** Baseline characteristics of participants.

Parameter	Data
Number of participants (*n*)	60
Average age (years) (mean ± SD)	33.3 ± 7.1
Mid‐frontal line (cm) (mean ± SD)	7.48 ± 0.46
Intertemporal line (cm) (mean ± SD)	14.66 ± 0.65

### 3.2 General Surgical Data

In the hair transplantation procedures, participants received an average of 3243 ± 1038 FUs, with a follicular density in the recipient area ranging from 50 to 60 FUs/cm^2^. The mean operative time was approximately 7.0 ± 2.1 h. Postoperatively, the mid‐frontal hairline was lowered by an average of 1.33 ± 1.10 cm, while the intertemporal distance was reduced by 1.47 ± 0.57 cm (Table 2).

**TABLE 2:** Summary of hair transplantation procedure details.

Parameter	Data
Average number of grafts (FUs) (mean ± SD)	3243 ± 1038
Follicular unit density in recipient area (FUs/cm^2^)	50–60
Average duration of surgery (h) (mean ± SD)	7.0 ± 2.1
Mid‐frontal line reduction (cm) (mean ± SD)	1.33 ± 1.10
Intertemporal line reduction (cm) (mean ± SD)	1.47 ± 0.57

### 3.3 Patient Satisfaction Assessment

**FIGURE 8:**
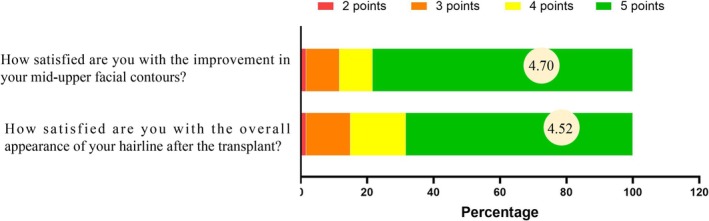
Distribution of patient‐reported satisfaction levels using a 5‐point Likert scale (1 = very dissatisfied, 2 = dissatisfied, 3 = neutral, 4 = satisfied, 5 = very satisfied). The mean satisfaction score regarding the mid‐upper facial contour improvement reached 4.70, and the overall satisfaction with the postoperative hairline appearance averaged 4.52.

According to the 5‐point Likert scale results, the majority of patients expressed satisfaction with the surgical outcomes. Regarding the improvement of the mid‐upper facial contours, the mean satisfaction score was 4.70. Specifically, 47 patients rated themselves as very satisfied, 6 as satisfied, 6 as neutral, and 1 as dissatisfied, with no reports of being very dissatisfied. As for the overall aesthetic appearance of the postoperative hairline, the mean satisfaction score reached 4.52. Among them, 41 patients were very satisfied, 10 patients were satisfied, 8 patients provided neutral feedback, and 1 patient was dissatisfied, with no reports of extreme dissatisfaction (Figure 8 and Table 3).

**TABLE 3:** Patient satisfaction scores after hair transplantation.

Question	Likert scores					Mean rating
	1	2	3	4	5	
1. How satisfied are you with the improvement in your mid‐upper facial contours?	0	1	6	6	47	4.70
2. How satisfied are you with the overall appearance of your hairline after the transplant?	0	1	8	10	41	4.52

### 3.4 Comparison of Postoperative Adverse Events

As summarized in Table 4, postoperative complications following FUE surgery included scalp itching in 4 patients, localized swelling in 1 patient, pain or discomfort in 1 patient, and folliculitis in 7 patients. No cases of low graft survival rate, postoperative infection, significant scarring, bleeding at the surgical site, or abnormal hair growth direction were observed. Additionally, none of the patients developed severe complications.

**TABLE 4:** Postoperative complications following FUE surgery.

Adverse event	Number of cases
Low graft survival rate	0
Postoperative infection	0
Itching of the scalp	4
Localized swelling	1
Noticeable scarring	0
Bleeding at the surgical site	0
Pain or discomfort	1
Follicular inflammation (folliculitis)	7
Improper hair growth direction	0
None complications observed	47
Total	60

### 3.5 Representative Cases

Female cases were designed following the hair planning concept outlined in Figures 2 and 3, with individualized adjustments made according to each patient's specific characteristics.

#### 3.5.1 Case 1

**FIGURE 9:**
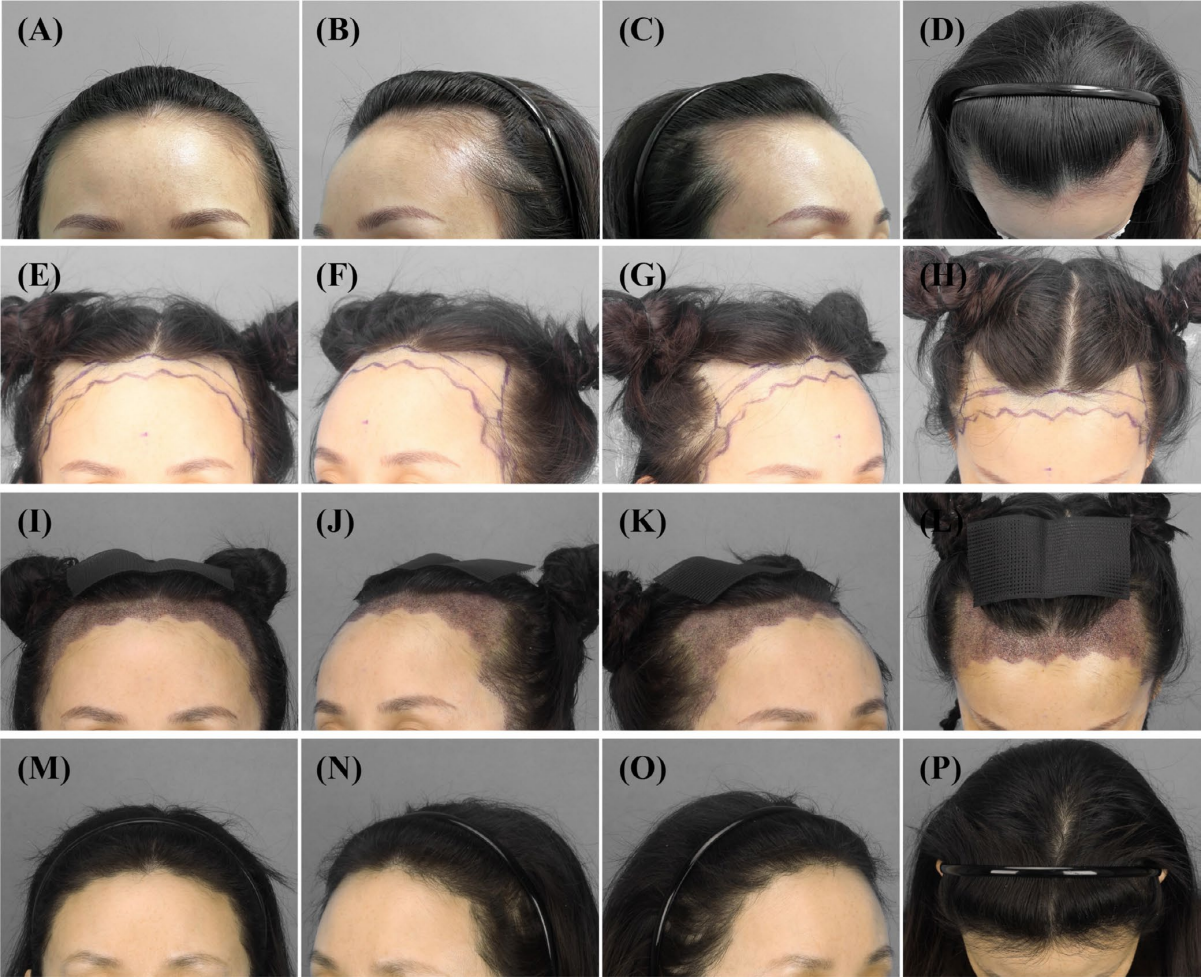
Clinical photographs of a 36‐year‐old female patient with a high and wide forehead, who underwent FUE transplantation with a total of 4000 grafts, consisting of 650 1‐hair thin units, 1200 1‐hair thick units, and 2150 2‐hair units. (A–D) Preoperative images; (E–H) preoperative design; (I–L) immediately after the surgery; (M–P) follow‐up at 13 months postoperatively.

A 36‐year‐old woman with a high and wide forehead underwent FUE hair transplantation based on the design concept in Figures 2 and 3. Follicular units containing 1, 2, or 3 hairs were harvested. During preparation, 3‐hair FUs were dissected into smaller units when needed. After dissection, grafts were classified and counted into 1‐hair thin units, 1‐hair thick units, and 2‐hair units based on the number of hairs and hair shaft diameter. Following separation and classification, the total number of 1‐hair thin units, 1‐hair thick units, and 2‐hair units was recorded and used as the final number of grafts. The transplantation area included key regions that influence the mid‐upper facial contour, namely the frontotemporal angle, midpoint of the frontal hairline, temporal peak, and sideburn areas. The implantation density was 60 units/cm^2^. A total of 4000 FUs were transplanted, including 650 1‐hair thin units, 1200 1‐hair thick units, and 2150 2‐hair units (Figure 9).

#### 3.5.2 Case 2

**FIGURE 10:**
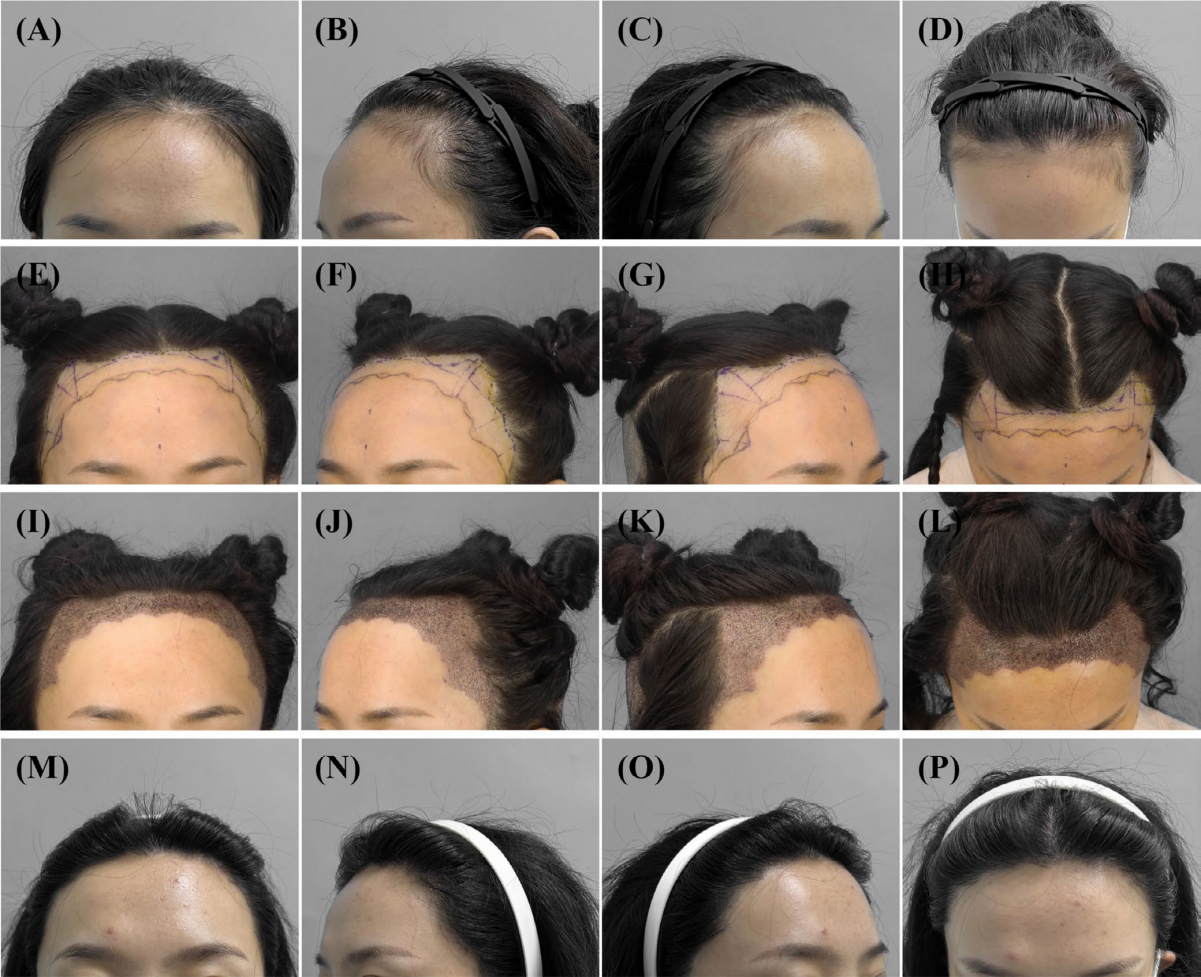
Clinical photographs of a 25‐year‐old female patient with a high and wide forehead, who underwent FUE transplantation with a total of 4500 grafts, consisting of 800 1‐hair thin units, 1350 1‐hair thick units, and 2350 2‐hair units. (A–D) Preoperative images; (E–H) preoperative design; (I–L) immediately after the surgery; (M–P) follow‐up at 10 months postoperatively.

A 25‐year‐old woman with a high and wide forehead similarly underwent FUE transplantation guided by the design principle illustrated in Figures 2 and 3. The surgical procedure and graft counting method were the same as in Case 1, with an implantation density of 60 units/cm^2^. A total of 4500 FUs were placed, consisting of 800 1‐hair thin units, 1350 1‐hair thick units, and 2350 2‐hair units (Figure 10).

## 4 Discussion

A high and wide forehead is often regarded as an incongruous factor in women's facial aesthetics. It tends to disrupt the facial proportions and weaken the three‐dimensionality of the mid‐upper facial contours, and results in a flattened and broadened appearance that can be perceived as aged or even masculine. Although these problems are mostly caused by congenital bone characteristics, women who are chronically affected by imbalance of forehead proportion may develop psychological burdens such as anxiety and low self‐esteem, ultimately impacting social confidence and quality of life [11].

Current methods for improving forehead contours include filler injection, forehead reduction surgery, and hair transplantation [3, 14, 15, 16]. Among these, hair transplantation not only directly reduces forehead height but also modifies the transverse width and has become one of the core treatment options [5, 11]. The surgical procedures are mainly categorized into follicular unit transplantation (FUT) and FUE. Compared with FUT, FUE does not involve strip excision of the scalp. It leaves no linear scar, offers faster recovery, and has become the most widely used donor harvesting method in clinical practice [17].

While FUE has become a widely accepted technique for donor harvesting, its application in female patients with high and wide foreheads should not focus solely on graft quantity or density. For East Asian women in particular—whose facial structures often feature a broad midface and flatter contours—the true value of hair transplantation lies in its capacity to reshape the mid‐upper face through precise aesthetic design.

This study centers on the hypothesis that strategic hairline reconstruction can significantly enhance the proportions of the mid‐upper facial region, which includes the vertical dimension of the forehead and the horizontal balance of the temporal region. Rather than merely covering bare skin, our approach defines the hairline as a facial framing structure that visually shortens the forehead, narrows the upper face, and enhances overall facial harmony. Compared to the vertex region, refining the frontal and lateral hairlines plays a more critical role in enhancing overall facial dimensionality [5, 11]. In practice, key aesthetic subunits such as the midpoint of the frontal hairline, frontotemporal recess, temporal peak, and sideburn region are redesigned with precise density gradients and curvature to create a soft, feminine, and proportional contour.

Compared with conventional hair transplantation for treating androgenetic alopecia, hairline transplant surgery for Asian women with high and wide foreheads has fundamentally different objectives and design principles. East Asian individuals often prefer a smaller facial appearance with a narrower forehead, aiming to enhance the overall balance of the mid‐upper face. By reducing both the height and width of the forehead through strategic hairline design, the mid‐upper facial contour appears shorter, more compact, and harmonious with East Asian aesthetic ideals. Rather than restoring hair loss in balding zones, the goal is to reshape the facial frame and reduce forehead prominence, thereby improving the aesthetic proportions of the mid‐upper face. This is particularly important in Asian women, who often have broader foreheads, lower hair density at the frontal hairline, and flatter midface contours. These anatomical features necessitate a customized hairline design, emphasizing the use of 1‐hair thin follicular units for a soft and feminine appearance, precise control of implantation angles, and strategic repositioning of the temporal peak to minimize the transverse facial width. These characteristics distinguish this procedure from standard FUE for hair loss, reinforcing its identity as a facial contouring solution rather than a density restoration technique.

The ideal vertical height of the face is typically divided into three equal parts: the upper third extends from the midpoint of the frontal hairline to the glabella, the middle third from the glabella to the subnasale, and the lower third from the subnasale to the menton [18]. If the upper third is significantly elongated, resulting in the distance from the midpoint of the frontal hairline to the glabella exceeding that of the middle third and lower third, it is considered a “high forehead” [3]. The ideal forehead height for women is usually 6–7 cm, and if it exceeds this range, it visually creates the impression that the forehead is too high [19]. Accordingly, in surgical design, the midpoint of the frontal hairline is usually positioned 6–7 cm above the glabella. Moreover, in patients with an M‐shaped hairline, the forehead often appears higher than in those with a rounded contour, even if the vertical height from the glabella tothe midpoint of the frontal hairline is the same [7, 20]. Therefore, filling the frontotemporal angle and converting the M‐shaped hairline to a rounded one by implantation of hair follicles not only enhances the naturalness but also further improves the visual proportion of the forehead. A wide midface is a common aesthetic concern in East Asian populations. Surgical correction of the lateral hairline by reconstructing the temporal peak and sideburn region can be an effective approach to reduce the horizontal width of the midface [5].

Hairline restoration should be strictly matched to the angle, direction, and density of hair growth according to the needs of different areas. Aesthetic refinement is achieved by selectively implanting 1‐hair thin units, 1‐hair thick units, and 2‐hair units to mimic the natural layering and density of normal scalp. The core of the aesthetic result lies in creating a seamless transition along the hairline. At the anterior edge, 1‐hair thin units are prioritized to outline a soft, curved, and feminine wavy hairline contour [21]. The approximately 2 cm wide transition zone immediately behind the anterior edge of the hairline is subdivided into two parts: the anterior half is implanted with 1‐hair thick units, while the posterior half uses a combination of 1‐hair thick units and 2‐hair units to form a smooth density gradient from the leading edge to the inner part, avoiding abrupt demarcation. To further enhance the fullness and natural layering of the hairline, 2‐hair units are placed in the frontotemporal angle and junction areas between native and transplanted hair.

During hair transplantation, the implantation of follicular units can also contribute to soft tissue augmentation in the forehead region. This effect is particularly evident in areas such as the temporal zone, where significant soft tissue loss occurs during aging [22]. In addition to reshaping facial contours, hair transplantation helps restore soft tissue volume, thereby promoting facial rejuvenation [23]. Moreover, beyond the direct volumizing effect, the perifollicular soft tissue contains abundant stem cells [24, 25], which may exert sustained paracrine activity after transplantation [26], potentially contributing to long‐term improvement in the aging skin condition of the frontal and temporal areas.

Although our team has achieved favorable outcomes in FUE hair transplantation for women with high and wide foreheads, this procedure imposes higher demands on the surgeon's aesthetic judgment, technical details, and team coordination. Ensuring a natural and lasting postoperative result requires specialized training and extensive clinical experience.

In terms of research limitations, there is a lack of multicenter, large‐sample prospective studies on hair transplantation for women with high and wide foreheads. In the future, there is an urgent need to expand the sample size to include women of different races and facial features, and to systematically evaluate the follicle survival rate, hair growth effect, and aesthetic improvement, in order to validate the broad applicability of the personalized design concept.

## 5 Conclusion

Hairline transplantation is a safe, effective, and aesthetically valuable treatment for correcting facial disproportion caused by a high and wide forehead in women. Through zonal surgical design and refined density distribution strategies, the procedure can effectively lower forehead height, reduce frontal width, and significantly enhance the mid‐upper facial contour and feminine features. Postoperative satisfaction regarding facial contour improvement and hairline naturalness was high, with a low incidence of complications and stable overall outcomes, indicating promising clinical applicability of this technique in East Asian female populations with high and wide foreheads.

## Author Contributions

Wei Wu and Chi Liu conceived and designed the study. Clinical data collection was performed by Wei Wu, Qian Liu, and Meihua Di. Data management and statistical analysis were conducted by Peiqi Zhang and Shunuo Zhang. Xiaohui Yang and Siyuan Zhu contributed to data interpretation and provided critical input. The manuscript was drafted by Wei Wu and Chi Liu with substantial contributions from all authors. All authors reviewed, revised, and approved the final version of the manuscript for submission.

## Conflicts of Interest

The authors declare no conflicts of interest.

## Data Availability

The data that support the findings of this study are available from the corresponding author upon reasonable request.

## References

[1] H. Jagadish Chandra , M. S. Ravi , S. M. Sharma , and B. Rajendra Prasad , “Standards of Facial Esthetics: An Anthropometric Study,” Journal of Maxillofacial and Oral Surgery 11, no. 4 (2012): 384–389. 24293927 10.1007/s12663-012-0355-9 PMC3485457

[2] L. G. Farkas and J. C. Kolar , “Anthropometrics and Art in the Aesthetics of Women's Faces,” Clinics in Plastic Surgery 14, no. 4 (1987): 599–616. 3652607

[3] B. Berenguer , T. García , C. Lorca‐García , and M. San‐Basilio , “Aesthetic Forehead Reduction in Female Patients: Surgical Details and Analysis of Outcome,” Journal of Plastic, Reconstructive & Aesthetic Surgery 75, no. 1 (2022): 407–414. 10.1016/j.bjps.2021.06.002 34305024

[4] B. Guyuron , R. A. Behmand , and R. Green , “Shortening of the Long Forehead,” Plastic and Reconstructive Surgery 103, no. 1 (1999): 218–223. 9915187 10.1097/00006534-199901000-00036

[5] J. H. Park , “Side‐Hairline Correction in Korean Female Patients,” Plastic and Reconstructive Surgery. Global Open 3, no. 3 (2015): e336. 25878947 10.1097/GOX.0000000000000304 PMC4387158

[6] S. C. Rhee and S. H. Lee , “Attractive Composite Faces of Different Races,” Aesthetic Plastic Surgery 34, no. 6 (2010): 800–801. 20953953 10.1007/s00266-010-9606-7

[7] J. H. Jung , D. K. Rah , and I. S. Yun , “Classification of the Female Hairline and Refined Hairline Correction Techniques for Asian Women,” Dermatologic Surgery 37, no. 4 (2011): 495–500. 21388483 10.1111/j.1524-4725.2011.01910.x

[8] S. H. Lee , Y. H. Oh , S. Youn , and J. S. Lee , “Forehead Reduction Surgery via an Anterior Hairline Pretrichial Incision in Asians: A Review of 641 Cases,” Aesthetic Plastic Surgery 45, no. 4 (2021): 1551–1560. 33683382 10.1007/s00266-020-02103-4

[9] S. S. Kabaker and J. P. Champagne , “Hairline Lowering,” Facial Plastic Surgery Clinics of North America 21, no. 3 (2013): 479–486. 24017989 10.1016/j.fsc.2013.05.007

[10] P. de Azevedo Marques , “Forehead Reduction Surgery: Outcomes and Complications of 650 Cases in a Multiracial Population,” Aesthetic Plastic Surgery. (2025), 10.1007/s00266-025-04830-y. 40240586

[11] J. H. Park , S. H. You , and N. Kim , “Frontal Hairline Lowering With Hair Transplantation in Asian Women With High Foreheads,” International Journal of Dermatology 58, no. 3 (2019): 360–364. 30675719 10.1111/ijd.14388

[12] A. Garg and S. Garg , “Overview of Follicular Extraction,” Indian Journal of Plastic Surgery 54, no. 4 (2021): 456–462. 34984085 10.1055/s-0041-1739244 PMC8719976

[13] K. Kashiyama , R. Haraguchi , F. Ban , et al., “Study of Frontal and Temporal Hairline Patterns in Japanese Subjects,” Plastic and Reconstructive Surgery. Global Open 9, no. 8 (2021): e3751. 34414058 10.1097/GOX.0000000000003751 PMC8367035

[14] P. M. Vila , S. N. Somani , Q. E. Wafford , and D. M. Sidle , “Forehead Reduction: A Systematic Review and Meta‐Analysis of Outcomes,” Facial Plastic Surgery & Aesthetic Medicine 24, no. 1 (2022): 34–40. 33601981 10.1089/fpsam.2020.0474

[15] J. H. Park , “Novel Principles and Techniques to Create a Natural Design in Female Hairline Correction Surgery,” Plastic and Reconstructive Surgery. Global Open 3, no. 12 (2015): e589. 26894014 10.1097/GOX.0000000000000548 PMC4727698

[16] S. R. Del Cueto , F. U. Galvez , A. Gritti , N. Kefalas , C. de la Guardia , and G. Kerson , “An Evaluation of VYC‐17.5L for Forehead Contouring: A Prospective, Open‐Label, Post‐Marketing Study,” Journal of Cosmetic Dermatology 24, no. 3 (2025): e70093. 40026273 10.1111/jocd.70093 PMC11874166

[17] F. Jimenez , M. Alam , J. E. Vogel , and M. Avram , “Hair Transplantation: Basic Overview,” Journal of the American Academy of Dermatology 85, no. 4 (2021): 803–814. 33905785 10.1016/j.jaad.2021.03.124

[18] F. G. Garritano and V. C. Quatela , “Surgical Anatomy of the Upper Face and Forehead,” Facial Plastic Surgery 34, no. 2 (2018): 109–113. 29631278 10.1055/s-0038-1637727

[19] J. H. Park and B. S. Suh , “Hairline Correction by Hair Transplantation for Reducing Apparent Face Length in Long‐Face Women,” Plastic and Reconstructive Surgery 151, no. 3 (2023): 511–519. 36730561 10.1097/PRS.0000000000009969

[20] W. R. Rassman , J. P. Pak , and J. Kim , “Phenotype of Normal Hairline Maturation,” Facial Plastic Surgery Clinics of North America 21, no. 3 (2013): 317–324. 24017973 10.1016/j.fsc.2013.04.001

[21] R. Shapiro , “Principles and Techniques Used to Create a Natural Hairline in Surgical Hair Restoration,” Facial Plastic Surgery Clinics of North America 12, no. 2 (2004): 201–217. 15135130 10.1016/j.fsc.2003.12.004

[22] R. Haidar , M. D. D. Freytag , K. Frank , et al., “Quantitative Analysis of the Lifting Effect of Facial Soft‐Tissue Filler Injections,” Plastic and Reconstructive Surgery 147, no. 5 (2021): 765e–776e. 10.1097/PRS.0000000000007857 33890889

[23] U. Wollina , “Facial Rejuvenation Starts in the Midface: Three‐Dimensional Volumetric Facial Rejuvenation Has Beneficial Effects on Nontreated Neighboring Esthetic Units,” Journal of Cosmetic Dermatology 15, no. 1 (2016): 82–88. 26304759 10.1111/jocd.12175

[24] A. Rezza , R. Sennett , M. Tanguy , C. Clavel , and M. Rendl , “PDGF Signalling in the Dermis and in Dermal Condensates Is Dispensable for Hair Follicle Induction and Formation,” Experimental Dermatology 24, no. 6 (2015): 468–470. 25708924 10.1111/exd.12672 PMC4943754

[25] C. F. Huang , Y. J. Chang , Y. Y. Hsueh , et al., “Assembling Composite Dermal Papilla Spheres With Adipose‐Derived Stem Cells to Enhance Hair Follicle Induction,” Scientific Reports 6 (2016): 26436. 27210831 10.1038/srep26436 PMC4876394

[26] C. L. Chen , W. Y. Huang , E. H. C. Wang , K. Y. Tai , and S. J. Lin , “Functional Complexity of Hair Follicle Stem Cell Niche and Therapeutic Targeting of Niche Dysfunction for Hair Regeneration,” Journal of Biomedical Science 27, no. 1 (2020): 43. 32171310 10.1186/s12929-020-0624-8 PMC7073016

